# Implementing an Eye Movement and Desensitization Reprocessing Treatment-Program for Women With Posttraumatic Stress Disorder After Childbirth

**DOI:** 10.3389/fpsyg.2021.797901

**Published:** 2022-01-21

**Authors:** Leonieke W. Kranenburg, Hilmar H. Bijma, Alex J. Eggink, Esther M. Knijff, Mijke P. Lambregtse-van den Berg

**Affiliations:** ^1^Department of Psychiatry, Erasmus University Medical Center, Rotterdam, Netherlands; ^2^Department of Obstetrics and Gynecology, Erasmus University Medical Center, Rotterdam, Netherlands; ^3^Department of Child & Adolescent Psychiatry, Erasmus University Medical Center, Rotterdam, Netherlands

**Keywords:** EMDR, women mental health, implementation, childbirth, PTSD – posttraumatic stress disorder, treatment program

## Abstract

**Purpose:**

The purpose of this study is to describe the implementation and outcomes of an Eye Movement and Desensitization Reprocessing (EMDR) treatment-program for women with posttraumatic stress disorder (PTSD) after childbirth.

**Methods:**

A prospective cohort-study with pre- and post-measurements was carried out in the setting of an academic hospital in the Netherland. Included were women who gave birth to a living child at least 4 weeks ago, with a diagnosis of PTSD, or severe symptoms of PTSD combined with another psychiatric diagnosis. All received up to 8 sessions of EMDR-therapy. The posttraumatic stress disorder Checklist for DSM-5 was administered before and after treatment. Trauma history was assessed before treatment with the Life Events Checklist for the DSM-5, the Childhood Trauma Questionnaire and the Childbirth Perception Scale. Descriptive statistics were used.

**Results:**

Forty-four women were referred, 26 met the inclusion criteria. After treatment, none of the women met the criteria for diagnosis of PTSD after on average 5 weekly sessions of EMDR- therapy. These outcomes are promising, as they were achieved in women with relatively high levels of psychiatric comorbidity (64%) and high rates of previous mental health treatment (80%).

**Conclusion:**

Implementing an EMDR-treatment program for women with PTSD after childbirth in the setting of an academic hospital is feasible and effective. Key factors for success include a close collaboration between the relevant hospital departments and a thorough case conceptualization addressing the etiology of the PTSD.

## Introduction

Posttraumatic stress disorder (PTSD) following childbirth occurs relatively frequent. Prevalence rates range from 3% in community samples and 15% in at risk populations (Grekin and O’Hara, 2014). PTSD after childbirth is most typically related to a traumatic delivery (Ayers et al., 2016) and is characterized by the re-experiencing of the traumatic event, avoidance, negative changes in mood and cognition and hyper arousal (APA, 2014). PTSD after childbirth not only negatively affects the mother’s health and the partner-relationship (Ayers et al., 2006), but also child outcomes. Maternal PTSD has a negative impact on the development and sensitivity of the stress-system in the infant, the mother-child bond, the attachment style of the child, and the child’s social-emotional and cognitive development (Ammerman et al., 2012; Parfitt et al., 2014; Garthus-Niegel et al., 2017, 2018; Cook et al., 2018). To reduce the mother’s disease burden and to prevent transgenerational transmission of mental health problems, treatment is warranted as soon as possible. Moreover, starting a new pregnancy with untreated PTSD has shown to be related to unfavorable fetal development and obstetric outcomes, poor maternal well-being, fear of childbirth, avoidance of pregnancy care and maternal requests for cesarean section, and therefore treatment should be initiated before a subsequent pregnancy (Stramrood et al., 2012; Baas et al., 2017; Nesari et al., 2018). In addition, treating women with severe PTSD symptoms who do not fulfill all diagnostic criteria should also be considered (Ayers et al., 2008; Verreault et al., 2012) because untreated PTSD symptoms after childbirth may lead to a chronic disorder (Baas et al., 2017; Yildiz et al., 2017; Dikmen-Yildiz et al., 2018). A recent review indeed underlined that untreated perinatal PTSD impacts long-term maternal and child health and contributes to health disparities (Small et al., 2020). Altogether, this makes a strong case for early recognition and referral for evidence-based treatment of PTSD in women after giving birth. So far however, data on treatment programs for this group are limited and indeed recent studies emphasize the need for exploration of effective interventions for perinatal PTSD in mothers (de Bruijn et al., 2020; Grekin et al., 2021).

Eye Movement and Desensitization and Reprocessing (EMDR)-therapy is an evidence-based treatment for PTSD and recommended in international guidelines, for example those of the WHO (WHO, 2013; Shapiro, 2014; NICE-guidelines, 2018). EMDR-therapy is effective in treating PTSD, with large effect sizes compared to control conditions, and comparable effects compared to cognitive behavioral therapies (Cuijpers et al., 2020). In a recent review we showed that EMDR- therapy may be a promising intervention for women with PTSD following childbirth (de Bruijn et al., 2020). However, referral for such treatment may be impeded for several reasons (van Dinter-Douma et al., 2020), such as poor recognition, ideas on that the first period after delivery would be too burdensome to start treatment, insecurity about the safety of EMDR-treatment during an already subsequent pregnancy, or lack of a structure for efficient referral for treatment. Therefore, the aim of this study is to investigate the feasibility of an EMDR-therapy program for women with PTSD following child birth and to evaluate the outcomes of such treatment.

## Materials and Methods

### Design

The current study was an observational prospective cohort-study with pre- and post measurements. The study was approved by the medical scientific research Ethical Committee of the Erasmus University Medical Centre and evaluated as exempt (reference number MEC-2018-1234). Study inclusion took place from January 2019 to June 2020. All participants gave written informed consent. No external funding was obtained for this study.

### Participants

Women suspected of PTSD following childbirth were recruited at three different departments of the Erasmus MC, a large academic hospital in Rotterdam, the Netherland: the department of Obstetrics and Gynecology, the department of Psychiatry and the department of Child and Adolescent Psychiatry. All physicians from those departments could refer women suspect of PTSD following childbirth for the current study. In case of doubt or questions about referral, physicians could consult the colleagues of the Psychiatry department by email or direct phone line. As our aim was to the study the feasibility of implementing an EMDR-treatment program for women with PTSD following childbirth in clinical practice, we stayed as close as possible to real-life referral practice. Therefore, all new patients referred were enrolled consecutively in this study and as such we made no exceptions. In accordance with this aim, the inclusion criteria were: giving birth to a living baby at least 4 weeks ago; a current PTSD diagnosis, or actual severe PTSD-symptoms combined with another DSM-5 diagnosis; and written informed consent. Exclusion criteria were: insufficient understanding of Dutch/English language, <18 years of age, (other) severe psychopathology that would require immediate treatment first, for example high suicidality risk or active psychosis.

### Procedures

Before the start of this study, members of the Psychiatry department (LK, MLvdB, and EK) provided clinical lessons on PTSD following childbirth for the colleagues of the department of Obstetrics and Gynecology. During these meetings, attention was paid to recognizing PTSD symptoms in women who recently gave birth. In addition, clinical training was given on how to discuss these symptoms and the possibilities for treatment. Education was given on how to pose the two most important questions in this respect: “Have you experienced any event during pregnancy, delivery or childbed period that you would describe as extremely stressful?” and 2. “If so, are you still suffering from this? For instance, do you have nightmares about what happened, or do you avoid talking/thinking about what has happened? Are you constantly alert as if something bad is about to happen?” To further enhance the screening process on PTSD after childbirth, screening questions were incorporated in the standard Patient Related Outcome Measures (PROMs)-assessment of women in the perinatal trajectory as part of value-based healthcare. In case women answered positive on these screening questions, outcomes were discussed during the following consultation with their gynecologists and obstetricians. Healthcare providers of the departments of Psychiatry received no clinical lessons, but were actively informed about this study during regular weekly team meetings in which treatment advice for women presenting with psychiatric complaints was decided upon. Women who seemed eligible for study participation and treatment could then be referred. The department of Child and Adolescent Psychiatry offers a so-called mother-child treatment program, focusing on mother-child interaction and bonding in women with perinatal psychiatric disorders. As one reason for impaired mother-child interaction is PTSD after childbirth in the mother, this department was informed about the study as well. Referred women were invited for an intake at the outpatient clinic of the Psychiatry department. Intakes were performed by a senior health care psychologist (LK) and psychiatrist specialized in the field of perinatal psychiatry (MLvdB). During intake, current psychopathology was evaluated, PTSD was assessed by systematically addressing PTSD symptoms according to the DSM-5 criteria, a DSM-5 classification was established and questionnaires were administered (see below, measures). If women met the inclusion criteria and gave informed consent, EMDR- therapy was offered (see below, intervention).

### Measurements

All questionnaires were administered at baseline. The PCL-5 was administered both before and after treatment.

#### Demographic Data

Age, previous and current psychopathology and obstetric data were collected at the moment of intake or were retrieved from the already present patient hospital records.

#### Trauma History and Posttraumatic Stress Disorder Symptoms

##### Posttraumatic Stress Disorder Checklist for the DSM-5 and Life Events Checklist for DSM-5 With Extended a Criterion

The LEC-5 (Weathers et al., 2013; Dutch Version: Boeschoten et al., 2014) is a self-report questionnaire to screen for 17 lifetime potentially traumatic events. Respondents indicate whether they have experienced one or more of sixteen listed events. The last item consists of an additional question, where respondents can indicate whether they have experienced a stressful event, other than the events mentioned in the previous items. Items are scored with regard to the type of exposure: direct experience; witnessing the trauma; learning that a traumatic event has happened to a close family member or friend and; experiencing a traumatic event as a part of the daily job. The PCL-5 is a widely used and well validated 20-item self-report questionnaire assessing the 20 symptoms of PTSD according to DSM-5. Respondents report the level of PTSD symptoms that they have experienced in the past month. Items are scored on a scale from 0 (not at all) to 4 (extremely). Scores range from 0–80, with higher scores representing more PTSD symptoms. The test-retest reliability of the total score of the PCL-5 is good (*r* = 0.82). Convergent and discriminant validity are strong (*rs* 0.74 to 0.85 and 0.31 to 0.60, respectively) (Blevins et al., 2015). A cut-off score of 31 is often used as indicator for the presence of PTSD (Bovin et al., 2016) and a 10–20 point change on this scale is perceived as clinically significant (Weathers et al., 2013).

##### Childhood Trauma Questionnaire-Short Form

This widely used self-reported childhood trauma questionnaire of 25 items (Bernstein et al., 2003) is the short version of the original CTQ (70 items). It is used to investigate five types of traumatic experiences of neglect (physical and emotional) and abuse (physical, emotional and sexual) in childhood and adolescence. CTQ-SF items are rated on a 5-point scale, ranging from 1 (never true) to 5 (very often true). Items 2, 5, 7, 12, 17, 23, and 25 are scored in reverse. Scores range from 25–125, with higher scores indicating more trauma experiences. Reliability coefficients (Cronbach’s alpha) of the subscales range from 0.61 to 0.95 (Bernstein et al., 2003).

##### Childbirth Perception Scale

The 12-item CPS (Truijens et al., 2014) consists of two dimensions, namely the perception of delivery and the 1st week after delivery, both measured with six items. Example statements from both categories are: “*My labor was a lot worse than I expected*” and “*I truly enjoyed the first week after delivery*”. All items are scored on a 4-point scale from 0 (fully agree) to 3 (completely disagree). Items 1, 2, 5, 6, 7, 8, and 10 are scored in reverse. Scores range from 0 to 36, with higher scores indicating a more adverse perception toward childbirth. The total scale, as well as both subscales, have a good reliability (Cronbach’s alphas >0.75) (Truijens et al., 2014).

### Intervention: Eye Movement and Desensitization Reprocessing Therapy

All women received up to eight weekly 90-min sessions of EMDR-therapy in the context of this study. The first session was allocated for case conceptualization. During this session, LEC-5, PCL-5, CTQ-SF, and CPS outcomes were thoroughly discussed. The reason for this is that previous trauma, be it pregnancy-related or not, can influence the development and persisting of actual PTSD symptoms. Based on this case-conceptualization, the course of treatment was designed. In session 2–7, women received EMDR-therapy following the 2020 version of the Dutch EMDR protocol (De Jongh and Ten Broeke, 2018). During each session, the target images, cognitive domains, the validity of (positive) cognitions (lowest and highest score), the subjective unit of distress (lowest and highest scores) were registered. Targets images refer to specific disturbing memory images of the traumatic event. A cognitive domain refers to the type of cognitions that make that a specific memory image still causes distress in the present, even though the event belongs to the past and even though the event may have had a good ending after all. The cognitive domains as applied in the Dutch EMDR protocol are: control, safety, self-evaluation and guilt. For instance, a memory image can have high load on the domain “self-evaluation”, if negative cognitions about the self are most prominent when a woman is confronted with the disturbing memory image. Session 8 consisted of an evaluation of treatment. If symptoms diminished and there was loss of diagnosis before session 8, treatment ended. If after session 8 symptoms persisted, the treatment plan was adjusted and women were offered appropriate continuation of treatment. Treatment was performed or supervised by a licensed EMDR Europe practitioner.

### Data-Analysis

Data were analyzed by means of descriptive statistics (M,SD) in IBM SPSS statistics (version 25). To calculate pre-post differences for PCL-5 outcomes, a dependent samples *t*-test was applied.

## Results

Forty-four women were referred. For all women referred, psychiatric treatment was indicated and offered. However, for the results presented below, only data from women who met the inclusion criteria are presented. Main reasons for exclusion were based on psychiatric assessment, in that another psychiatric disorder (than PTSD) was more prominent and/or required treatment first. Twenty-six women were included and 25 completed treatment. We had one drop-out due to severe family circumstances, with unexpected illness and death of a close family member during the first COVID-19 outbreak, which made continuation of EMDR-therapy not possible for her at the time. Respondent characteristics and main outcomes are presented in Table 1. Mean age was 32, and women were referred on average 10 months after giving birth. In most cases there was a comorbid psychiatric disorder present, most often depression. Most women had received mental health treatment earlier in life. Almost all women had experienced (other) traumatic events in the past, as is shown by their scores on the LEC-5 and CTQ. There was a statistically significant difference in the PCL-5 score before (M 46.33, SD 14.19) and after treatment (M 14.58, SD 11.97), *t*(23) = 9,835, *p* = 0.000.

**TABLE 1:** Respondent characteristics and main outcomes.

	*N* = 25 women
**Age (mean, SD)**	32.36 (4.56)
**Months since delivery (mean, range)**	10 (2–42)
**Number of children**	
1	13
2	5
3	2
4	1
Pregnant (2nd or 3rd child)	4
**Previous mental health problems** (%, n)	80% (20/25)
**Psychiatric comorbidity** (%, n)	64% (16/25)
Depressive disorder (%, n)	52% (13/25)
**LEC-5 no. of events** (mean, SD)	5.08 (2.41)
**CTQ-total score** (mean, SD)*	33.61 (10.29)
CTQ-emotional abuse (%,n above cut-off)^†^	26% (6)
CTQ-physical abuse (%,n above cut-off)	9% (2)
CTQ-sexual abuse (%, n above cut-off)	9% (2)
CTQ-emotional neglect (%,n above cut-off)	39% (9)
CTQ-physical neglect (%,n above cut-off)	17% (4)
**CPS-total score** (mean, SD)*	24.32 (6.58)
CPS-delivery (mean, SD)	10.73 (4.47)
CPS-1st week postpartum (mean, SD)	13.59 (3.29)
**PCL-5 score baseline** (mean, SD)	44.84 (15.77)
**PCL-5 score after treatment** (mean, SD)*	14.58 (11.97)

*Missings were excluded. There were 2 missings for the CTQ, 3 for the CPS and 1 for PCL-score after treatment.

*^†^The CTQ-cut-off scores for low severity abuse were used (Spinhoven et al., 2014).*

Table 2 shows the treatment specific characteristics. Average treatment duration was 4.96 (SD 3.67) sessions. All women lost their PTSD diagnosis. Per treatment, on average 3.12 (SD 2.37) “targets” were neutralized. The cognitive domain control was most common for the selected memory images.

**TABLE 2:** EMDR-therapy specific characteristics.

	*N* = 24 women
**Number of sessions** *(mean, SD)*	4.96 (3.67)
**Number of targets during treatment** *(mean, SD)*	3.12 (2.37)
**Cognitive domain**	
Control *(%), [*+ flashforwards* (%)]	59%, (21%)
Self-evaluation *(%)*	15%
Safety *(%)*	1%
Guilt *(%)*	4%

*“Flashforwards” are a special technique associated with the cognitive domain of control. Within the flashforward-technique, it is not the adverse memory that is targeted, but anxious expectations of what may happen in the future, so-called “disaster fantasies”.

## Discussion

All women in our study showed a major and clinically relevant decrease in PTSD symptoms after on average 5 weekly sessions of EMDR. The average decrease was 30 points on the PCL-5, whereas a decrease of 10–20 points on this scale is already considered clinically significant (Weathers et al., 2013). All women lost their PTSD diagnosis. These outcomes are extra promising, as they were achieved in women with relatively high levels of psychiatric comorbidity and high rates of previous mental health treatment.

In many women in our sample a comorbid depressive disorder was present. PTSD and depressive disorder often co-exist and interfere, and depression both during pregnancy and after childbirth influence the trauma response (Ayers et al., 2016; King et al., 2017). There is an overlap in symptoms between depression and PTSD (APA, 2014; Grekin et al., 2021), for instance with regard to negative changes in mood and cognition. King et al. (2017) found that negative cognitions about the self in relation to the birth were the strongest cognitive behavioral predictors of PTSD. These findings underscore the need to explicitly address feelings of shame, self-blame, guilt and responsibility in making an adequate plan for treatment. We indeed found that these types of emotions were common. Still, women may find it hard to acknowledge their distress and initially try to downplay or avoid their problems, until the point that they feel they have no other option than to seek help (Slade et al., 2021).

Another finding of the present study was that the cognitive domain of “control” was by far the most prevalent cognitive domain in explaining why certain memory images were still disturbing. This high prevalence of the cognitive domain “control” is in line with findings on the treatment of non-childbirth related PTSD (De Jongh and Ten Broeke, 2021). So, in this respect, PTSD following childbirth is comparable to “other PTSDs”. The high prevalence of the cognitive domain “control” makes sense conceptually, as pregnancy and childbirth are by definition situations where a certain unpredictably and loss of control are rather rule than exception.

### Clinical Implications

In our experience it is important to ensure bi-directional low-key options for consultation and advice between Psychiatry and Gynecology and Obstetrics departments, including regular interdisciplinary meetings. Moreover, we noticed that informing women on these lines of collaboration adds to the trust of the women in their treatment. Although women’s trust in treatment may increase the chance for successful treatment outcomes in general, trust is especially important in this specific group whose trust, in themselves or others, may have been violated. Further, with regard to the psychiatric treatment, it is recommended to pay close attention to previous trauma, as we did in our study by administering the CTQ and LEC-5-questionnaires. Although PTSD after childbirth can be the direct result of a pregnancy, birth or childbed-related event, pregnancy-related experiences can also trigger the memories of previous trauma, such as adverse sexual experience (Ayers et al., 2016; King et al., 2017). In this study, we started treatment with a thorough case conceptualization in collaboration with the women. In general, women were well able to indicate which symptoms were most burdensome, how these related (or not) to previous traumatic experiences, and consequently which complaints needed treatment first.

### Strengths and Limitations

A strength of the study is that it is driven by both current literature and clinical practice. Both perspectives acknowledge the need for adequate referral-and treatment lines for women with PTSD after childbirth. In this respect, our study fits within the current *Zeitgeist* by starting to fill a gap in literature. Our results provide a basis for future research and/or implementation of EMDR treatment programs in other hospitals. To our knowledge, the sample we describe is unique as it is the first in its kind describing EMDR outcomes for women with postpartum PTSD and high levels of psychiatric comorbidity. At the same time, such design holds the limitation of including a heterogeneous sample. Still we believe that these outcomes are valuable, because they resemble clinical practice. As such, these outcomes provide new insights on what to expect when starting hospital-based treatment program for women with PTSD following childbirth. Although the sample size of this study could be described as small, we believe that outcomes are convincing enough to positively answer the research question with regard to feasibility. A limitation of the current study is that a PTSD diagnosis was not made with a formal clinical interview such as the CAPS-5, which is the gold standard for making a clinical diagnosis for research purposes. Furthermore, although the results of this study are promising, the findings need confirmation of studies applying more advanced research design that include a control group. Future studies preferably also include outcome-measures for child outcomes and cost-effectiveness.

## Conclusion

Implementing an EMDR-therapy treatment program for women with PTSD after childbirth in the context of a large academic hospital is feasible and effective. Treatment led to clinically significant decrease of symptoms and loss of PTSD diagnosis in all cases. Results can be achieved in a short time-span, even in pregnant women and women with comorbid psychiatric disorders and/or a history of previous mental treatment. Key factors for success are a close collaboration between the relevant hospital departments and a thorough case conceptualization addressing the etiology of the PTSD after childbirth.

## Data Availability Statement

The data that support the findings of this study are available on request from the corresponding author.

## Ethics Statement

The studies involving human participants were reviewed and approved by Medical Scientific Research Ethical Committee of the Erasmus University Medical Centre. The patients/participants provided their written informed consent to participate in this study.

## Author Contributions

LK: conceptualization, data curation, formal analysis, investigation, methodology, validation, visualization, and writing – original draft. HB: data curation, investigation, and writing – review and editing. AE: conceptualization, investigation, and writing – review and editing. EK: conceptualization, investigation, and writing – review and editing. ML-V: conceptualization, data curation, investigation, methodology, and writing – review and editing. All authors contributed to the article and approved the submitted version.

## Conflict of Interest

The authors declare that the research was conducted in the absence of any commercial or financial relationships that could be construed as a potential conflict of interest.

## Publisher’s Note

All claims expressed in this article are solely those of the authors and do not necessarily represent those of their affiliated organizations, or those of the publisher, the editors and the reviewers. Any product that may be evaluated in this article, or claim that may be made by its manufacturer, is not guaranteed or endorsed by the publisher.

## References

[B1] Ammerman R. T. Putnam F. W. Chard K. M. Stevens J. Van Ginkel J. B. (2012). PTSD in depressed mothers in home visitation. *Psychol. Trauma* 4:186. 10.1037/a0023062 24307928 PMC3845816

[B2] APA (2014). *Handbook for the Classification of Mental Disorders (DSM-5). Dutch Translation of Diagnostic and Statistical Manual of Mental Disorders*, 5th Edn. Arlington: American Psychiatric Association.

[B3] Ayers S. Bond R. Bertullies S. Wijma K. (2016). The aetiology of post-traumatic stress following childbirth: a meta-analysis and theoretical framework. *Psychol. Med.* 46 1121–1134. 10.1017/S0033291715002706 26878223

[B4] Ayers S. Eagle A. Waring H. (2006). The effects of childbirth-related post-traumatic stress disorder on women and their relationships: a qualitative study. *Psychol. Health Med.* 11 389–398. 10.1080/13548500600708409 17129916

[B5] Ayers S. Joseph S. McKenzie-McHarg K. Slade P. Wijma K. (2008). Post-traumatic stress disorder following childbirth: current issues and recommendations for future research. *J. Psychosom. Obstet. Gynecol.* 29 240–250. 10.1080/01674820802034631 18608815

[B6] Baas M. A. M. Stramrood C. A. I. Dijksman L. M. de Jongh A. Van Pampus M. G. (2017). The OptiMUM-study: EMDR therapy in pregnant women with posttraumatic stress disorder after previous childbirth and pregnant women with fear of childbirth: design of a multicentre randomized controlled trial. *Eur. J. Psychotraumatol.* 8:1293315. 10.1080/20008198.2017.1293315 28348720 PMC5345578

[B7] Bernstein D. P. Stein J. A. Newcomb M. D. Walker E. Pogge D. Ahluvalia T. (2003). Development and validation of a brief screening version of the Childhood Trauma Questionnaire. *Child Abuse Negl.* 27 169–190. 10.1016/s0145-2134(02)00541-0 12615092

[B8] Blevins C. A. Weathers F. W. Davis M. T. Witte T. K. Domino J. L. (2015). The posttraumatic stress disorder checklist for DSM-5 (PCL-5): development and initial psychometric evaluation. *J. Trauma. Stress* 28 489–498. 10.1002/jts.22059 26606250

[B9] Boeschoten M. A. Bakker A. Jongedijk R. A. Olff M. (2014). *Checklist voor de DSM-5 (PCL-5) en Life Events Checklist voor de DSM-5 (LEC-5) met Uitgebreide A Criterium.* Diemen: Arq Psychotrauma Expert Groep.

[B10] Bovin M. J. Marx B. P. Weathers F. W. Gallagher M. W. Rodriguez P. Schnurr P. P. (2016). Psychometric properties of the PTSD checklist for diagnostic and statistical manual of mental disorders–fifth edition (PCL-5) in veterans. *Psychol. Assess.* 28:1379. 10.1037/pas0000254 26653052

[B11] Cook N. Ayers S. Horsch A. (2018). Maternal posttraumatic stress disorder during the perinatal period and child outcomes: a systematic review. *J. Affect. Disord.* 225 18–31. 10.1016/j.jad.2017.07.045 28777972

[B12] Cuijpers P. Veen S. C. Sijbrandij M. Yoder W. Cristea I. A. (2020). Eye movement desensitization and reprocessing for mental health problems: a systematic review and meta-analysis. *Cogn. Behav. Ther.* 49 165–180. 10.1080/16506073.2019.1703801 32043428

[B13] de Bruijn L. Stramrood C. A. Lambregtse-van den Berg M. P. Rius Ottenheim N. (2020). Treatment of posttraumatic stress disorder following childbirth. *J. Psychosom. Obstet. Gynecol.* 41 5–14. 10.1080/0167482X.2019.1593961 31164035

[B14] De Jongh A. Ten Broeke E. (2018). *Handboek EMDR: Een Geprotocolleerde Behandelmethode voor de Gevolgen van Psychotrauma (Handbook EMDR: A Protocolled Treatment for the Consequences of Psychotrauma)*, 7th Edn. Amsterdam: Pearson Assessment and Information B.V.

[B15] De Jongh A. Ten Broeke E. (2021). *EMDR Basic Course Training Material (Unpublished Material).*

[B16] Dikmen-Yildiz P. Ayers S. Phillips L. (2018). Longitudinal trajectories of post-traumatic stress disorder (PTSD) after birth and associated risk factors. *J. Affect. Disord.* 229 377–385. 10.1016/j.jad.2017.12.074 29331697

[B17] Garthus-Niegel S. Ayers S. Martini J. Von Soest T. Eberhard-Gran M. (2017). The impact of postpartum post-traumatic stress disorder symptoms on child development: a population-based, 2-year follow-up study. *Psychol. Med.* 47 161–170. 10.1017/S003329171600235X 27682188

[B18] Garthus-Niegel S. Horsch A. Ayers S. Junge-Hoffmeister J. Weidner K. Eberhard-Gran M. (2018). The influence of postpartum PTSD on breastfeeding: a longitudinal population-based study. *Birth* 45, 193–201. 10.1111/birt.12328 29265443

[B19] Grekin R. O’Hara M. W. (2014). Prevalence and risk factors of postpartum posttraumatic stress disorder: a meta-analysis. *Clin. Psychol. Rev.* 34 389–401. 24952134 10.1016/j.cpr.2014.05.003

[B20] Grekin R. O’Hara M. W. Brock R. L. (2021). A model of risk for perinatal posttraumatic stress symptoms. *Arch. Womens Ment. Health* 24 259–270. 32995950 10.1007/s00737-020-01068-2

[B21] King L. McKenzie-McHarg K. Horsch A. (2017). Testing a cognitive model to predict posttraumatic stress disorder following childbirth. *BMC Pregnancy Childbirth* 17:32. 10.1186/s12884-016-1194-3 28088194 PMC5237569

[B22] Nesari M. Olson J. K. Vandermeer B. Slater L. Olson D. M. (2018). Does a maternal history of abuse before pregnancy affect pregnancy outcomes? A systematic review with meta-analysis. *BMC Pregnancy Childbirth* 18:404. 10.1186/s12884-018-2030-8 30326858 PMC6192330

[B23] NICE-guidelines (2018). *Post-traumatic Stress Disorder NICE Guideline* [Online]. Available online at: www.nice.org.uk/guidance/ng116 (accessed May 5, 2021)

[B24] Parfitt Y. Pike A. Ayers S. (2014). Infant developmental outcomes: a family systems perspective. *Infant Child Dev.* 23 353–373.

[B25] Shapiro F. (2014). The role of eye movement desensitization and reprocessing (EMDR) therapy in medicine: addressing the psychological and physical symptoms stemming from adverse life experiences. *Perm. J.* 18:71. 10.7812/TPP/13-098 24626074 PMC3951033

[B26] Slade P. Molyneux R. Watt A. (2021). A systematic review of clinical effectiveness of psychological interventions to reduce post traumatic stress symptoms following childbirth and a meta-synthesis of facilitators and barriers to uptake of psychological care. *J. Affect. Disord.* 281 678–694. 10.1016/j.jad.2020.11.092 33220947

[B27] Small M. J. Gondwe K. W. Brown H. L. (2020). Post-traumatic stress disorder and severe maternal morbidity. *Obstet. Gynecol. Clin.* 47 453–461. 10.1016/j.ogc.2020.04.004 32762930

[B28] Spinhoven P. Penninx B. W. Hickendorff M. van Hemert A. M. Bernstein D. P. Elzinga B. M. (2014). Childhood Trauma Questionnaire: factor structure, measurement invariance, and validity across emotional disorders. *Psychol. Assess.* 26:717–729. 10.1037/pas0000002 24773037

[B29] Stramrood C. A. I. van der Velde J. Doornbos B. Paarlberg M. K. Weijmar Schultz W. C. M. van Pampus M. G. (2012). The patient observer: eye-movement desensitization and reprocessing for the treatment of posttraumatic stress following childbirth. *Birth* 39 70–76. 10.1111/j.1523-536X.2011.00517.x 22369608

[B30] Truijens S. E. M. Wijnen H. A. Pommer A. M. Oei S. G. Pop V. J. M. (2014). Development of the Childbirth Perception Scale (CPS): perception of delivery and the first postpartum week. *Arch. Womens Ment. Health* 17 411–421. 10.1007/s00737-014-0420-0 24663684

[B31] van Dinter-Douma E. E. de Vries N. E. Aarts-Greven M. Stramrood C. A. I. van Pampus M. G. (2020). Screening for trauma and anxiety recognition: knowledge, management and attitudes amongst gynecologists regarding women with fear of childbirth and postpartum posttraumatic stress disorder. *J. Matern. Fetal Neonatal Med.* 33 2759–2767. 10.1080/14767058.2018.1560409 30563384

[B32] Verreault N. Da Costa D. Marchand A. Ireland K. Banack H. Dritsa M. (2012). PTSD following childbirth: a prospective study of incidence and risk factors in Canadian women. *J. Psychosom. Res.* 73 257–263. 10.1016/j.jpsychores.2012.07.010 22980529

[B33] Weathers F. W. Litz B. T. Keane T. M. Palmieri P. A. Marx B. P. Schnurr P. P. (2013). *The PTSD Checklist for DSM-5 (PCL-5)* [Online]. Available online at: https://www.ptsd.va.gov/professional/assessment/adult-sr/ptsd-checklist.asp (accessed October 18, 2021)

[B34] WHO (2013). *WHO Releases Guidance on Mental Health Care after Trauma* [Online]. Geneva: WHO. 24344533

[B35] Yildiz P. D. Ayers S. Phillips L. (2017). The prevalence of posttraumatic stress disorder in pregnancy and after birth: a systematic review and meta-analysis. *J. Affect. Disord.* 208 634–645. 10.1016/j.jad.2016.10.009 27865585

